# Liposomal bupivacaine for popliteal sciatic and saphenous nerve blocks in patients undergoing foot and ankle surgery: a single-center, double-blind, randomized controlled trial

**DOI:** 10.3389/fmed.2026.1753354

**Published:** 2026-03-03

**Authors:** Jun Wang, Yan Yang, Fu-Qiang Chen, Kang Wang, Xue-Cheng Meng, Jian Zheng

**Affiliations:** Department of Anesthesiology, Xuzhou Renci Hospital, Xuzhou, Jiangsu, China

**Keywords:** foot and ankle surgery, liposomal bupivacaine, long-acting analgesia, popliteal sciatic nerve block, saphenous nerve block

## Abstract

**Background:**

Patients undergoing foot and ankle surgery often experience severe postoperative pain. This study aimed to assess the efficacy of liposomal bupivacaine for popliteal sciatic and saphenous nerve blocks in managing pain after foot and ankle surgery.

**Methods:**

The study was registered with the Chinese Clinical Trial Registry (ChiCTR2400088305) and received ethical approval from the Institutional Review Board of Xuzhou Renci Hospital (XZRCLL-KT-202407003). In this randomized trial, 142 patients undergoing elective foot/ankle surgery received popliteal-sciatic and saphenous nerve blocks with either 50 mg ropivacaine (R group) or 133 mg liposomal bupivacaine (L group). Primary outcome was postoperative sufentanil consumption; secondary outcomes included analgesia duration, motor blockade, recovery quality, sleep quality, and adverse events.

**Results:**

Compared with Group R, Group L demonstrated significantly lower sufentanil consumption at 12 h, 24 h, 48 h, and 72 h postoperatively (all *p* < 0.001). The L group exhibited significantly higher sleep quality (*p* < 0.001) and quality of recovery (*p* < 0.001) on postoperative day 1. Multivariable logistic regression analysis identified surgical type, preoperative sleep quality, and Pain Catastrophizing Scale scores as independent predictors of postoperative pain trajectory.

**Conclusion:**

The administration of liposomal bupivacaine for popliteal sciatic and saphenous nerve blocks significantly reduces postoperative opioid consumption and extends nerve block duration, providing a safe and effective technique for postoperative analgesia in patients undergoing foot and ankle surgery. Preoperative sleep quality, surgical type, and Pain Catastrophizing Scale score are independent predictors of the pain trajectory that can identify patients more likely to benefit from liposomal bupivacaine.

**Clinical trial registration:**

https://www.chictr.org.cn, Identifier ChiCTR2400088305.

## 1 Introduction

With continuous advancements and refinements in medical technology, treatment guidelines for foot and ankle injuries have been significantly improved. Comprehensive surgical protocols have now been established for various conditions, including fractures of the medial, lateral, and posterior malleoli; tibiofibular fractures; midfoot and forefoot fractures (such as metatarsal and phalangeal fractures); as well as ligamentous injuries (1, 2). Surgery serves as a vital tool for treating and preventing diseases, plays a critical role in fracture healing, joint reconstruction, and ligament repairs, and enhances patient quality of life (3, 4). However, patients with foot and ankle disorders frequently experience severe perioperative pain (5), if suboptimally managed, this may even lead to chronic pain, resulting in persistent articular discomfort and functional impairment (6).

Recognized as a primary complication of surgery, postoperative pain represents a substantial challenge for healthcare systems worldwide (7). Inadequate pain control not only triggers fluctuations in intraoperative blood pressure and heart rate but also, through a cascade of stress responses (8, 9), increases postoperative adverse events, prolongs hospital stays, and heightens the financial burden on patients. Studies indicate that the incidence of moderate to severe postoperative pain following orthopedic surgery exceeds 50% (10). As a critical subset of orthopedic procedures, foot and ankle surgery presents unique challenges due to the anatomically complex structure of the joint. Concurrent injuries to tendons, cartilage, and ligaments at fracture sites (11) further exacerbate patients’ postoperative pain. Thus, implementing targeted interventions to improve pain management in foot and ankle surgery patients is clinically imperative.

Peripheral nerve blocks provide effective analgesia and reduce the need for opioid analgesics, playing a critical role in postoperative pain management (12). Owing to the inherent pharmacological properties of local anesthetics, nerve blocks typically cannot provide prolonged analgesic effects (13). Consequently, strategies to extend the duration of analgesia have become a major research focus in recent years, aiming to better harness the clinical benefits of nerve blocks. Liposomal bupivacaine features a unique multivesicular structure that enables the slow release of encapsulated bupivacaine, thereby extending the duration of analgesia (14). Reportedly, liposomal bupivacaine can provide analgesic effects for up to 72 h (15), making it an emerging agent for perioperative pain management. Recent studies have demonstrated that liposomal bupivacaine, when administered via an interscalene brachial plexus block for shoulder surgery, significantly reduces postoperative pain compared with both placebo and standard bupivacaine (16). In thoracic surgery patients, intercostal nerve blocks utilizing liposomal bupivacaine have markedly prolonged the analgesic duration (17). Additionally, the administration of liposomal bupivacaine for transversus abdominis plane (TAP) blocks reduces opioid-related side effects in patients undergoing cesarean delivery and enhances postoperative recovery quality (18). However, no studies have investigated the application of liposomal bupivacaine in foot and ankle surgery populations.

Therefore, we conducted this randomized controlled trial to evaluate the efficacy of liposomal bupivacaine in patients undergoing foot and ankle surgery. Our primary hypothesis is that for patients undergoing foot and ankle surgery, popliteal sciatic and saphenous nerve blocks using liposomal bupivacaine will reduce postoperative opioid consumption and prolong analgesic efficacy. Second, we hypothesize that the use of liposomal bupivacaine will mitigate postoperative pain-related adverse effects and enhance postoperative recovery quality in this patient population.

## 2 Materials and methods

### 2.1 Study design and participants

We conducted this single-center, double-blind, randomized controlled trial with two parallel groups in accordance with the Consolidated Standards of Reporting Trials (CONSORT) statement. The study was registered with the Chinese Clinical Trial Registry (ChiCTR2400088305) and received ethical approval from the Institutional Review Board of Xuzhou Renci Hospital (XZRCLL-KT-202407003). All eligible patients who provided consent to undergo the treatment were required to submit written informed consent prior to randomization.

The inclusion criteria were as follows: BMI ranging from 18.5 to 35.0 kg/m^2^, aged 18 years or older, ASA I-III, and scheduled for Foot and Ankle surgery. The exclusion criteria were as follows: contraindications to nerve block anesthesia, pregnancy status, inability to cooperate, liver and kidney dysfunction, participation in other drug trials, allergy to research drugs, allergy to local anesthetics, infection at the puncture site, abnormal coagulation function, and incomplete case data.

### 2.2 Randomization, blinding and allocation concealment

The patients were randomly allocated at a 1:1 ratio to either the ropivacaine group (group R) or the liposomal bupivacaine group (group L) via a computer-generated random number table. The randomization details were securely stored in sequentially numbered, sealed opaque envelopes. Upon entering the operating room, a designated study pharmacist (medication preparer) opened the assigned envelope in the anesthesia preparation room to prepare the corresponding study medication according to group allocation.

To ensure blinding integrity, all prepared syringes containing liposomal bupivacaine were systematically wrapped in aluminum foil to conceal their characteristic opaque appearance. Following anesthesia induction, the study pharmacist delivered the prepared medication to the operating room anesthesiologist for peripheral nerve blockade administration. Importantly, this pharmacist had no involvement in any other aspects of the clinical trial beyond medication preparation.

A comprehensive double-blind protocol was maintained throughout the study period. All personnel involved in preoperative/postoperative assessments, surgical team members (including surgeons and nurses), outcome evaluators (including those assessing the duration of nerve blocks), and participating patients remained blinded to group assignments until final data analysis.

### 2.3 Intervention

Building on previous findings that 133 mg of liposomal bupivacaine provides effective analgesia and a favorable safety profile for peripheral nerve blocks, and based on the total local anesthetic requirement for combined sciatic and saphenous nerve blockade, we selected 133 mg as the dose for this study (18, 19). Similarly, patients in the control group received 20 mL of 0.25% ropivacaine (equivalent to 50 mg), a dosage selected based on previous literature and the findings of Wu et al. (20), which is widely recognized as an effective and safe clinical dose range. Accordingly, in the present study, patients in group L received 133 mg of liposomal bupivacaine diluted with normal saline to a total volume of 20 mL, while those in group R received 50 mg of 0.25% ropivacaine, also diluted with normal saline to 20 mL. To maintain the stability of liposomal bupivacaine, all dilution procedures were performed gently (21, 22). No adjuvants (e.g., dexamethasone or epinephrine) were added in any group to eliminate the potential influence of adjuvants on block characteristics and ensure the purity of comparison. Following the induction of standard general anesthesia and endotracheal intubation, all nerve blocks were performed using a standardized technique under ultrasound guidance. The attending anesthesiologists ensured satisfactory spread of the local anesthetics around the target neural structures, thereby ensuring comparability in block quality between the two groups.

During popliteal sciatic nerve block, with the assistance of the surgeon in lifting the lower limb, a high-frequency probe is used to scan superiorly to the popliteal crease. The popliteal artery and its accompanying superior popliteal vein were identified. The tibial nerve courses adjacent to the popliteal artery, whereas the common peroneal nerve lies lateral to the popliteal artery. When the probe is moved proximally, the common peroneal nerve progressively converges with the tibial nerve, ultimately forming the sciatic nerve. The target needle insertion site should be selected within the paraneural sheath of the sciatic nerve at the bifurcation gap where the nerve begins to divide. A 22-gauge needle was used for in-plane insertion. After confirming the absence of blood via aspiration, 15 mL of diluted solution from Group L and 15 mL of diluted solution from Group R were administered.

During saphenous nerve block, the patient is placed in a supine position. The ultrasound probe was then transversely placed over the anteromedial mid-thigh, perpendicular to the femur, to visualize the hyperechoic femoral bone. First, the femoral artery was identified, and then the probe was gently slid posteriorly. The saphenous nerve is an oval-shaped hyperechoic structure located superomedial to the femoral artery, beneath the inferior border of the sartorius muscle, and anterior to the adductor magnus muscle. A 22-gauge needle is advanced via the in-plane technique. After confirming that no blood returned upon aspiration, 5 mL of diluted solution was injected into Group L, and 5 mL of diluted solution was injected into Group R.

### 2.4 Postoperative pain management

On the day preceding surgery, informed consent was obtained from participants, and comprehensive education regarding patient-controlled analgesia (PCA) pump utilization was provided. The PCA solution was formulated as follows: 100 μg of sufentanil combined with 10 mg of tropisetron was diluted with normal saline to a total volume of 100 mL. The PCA parameters were configured as follows: continuous basal infusion at 1 mL/h, a bolus dose of 2 mL per activation, a lockout interval of 5 min, and a loading dose of 10 mL/h.

The PCA system was initiated during skin closure. Following extubation, patients were transferred to the postanaesthesia care unit (PACU) for continuous monitoring. Patients who achieved a Steward Recovery Score ≥ 4 after 30 min of PACU observation were subsequently transferred to the general ward. Throughout both the PACU and ward care, patients were instructed to activate the PCA bolus when they experienced pain intensity ≥ 3 on the visual analog scale (VAS). In instances of inadequate pain relief (VAS score ≥ 3 persisting for >30 min despite PCA use), rescue analgesia with intravenous tramadol 50 mg was administered.

### 2.5 Surgical procedure

The surgical procedures included in this study were categorized into two groups: fracture-related interventions and nonfracture injury management. Fracture-related procedures primarily included ankle fractures, tibiofibular fractures, and foot fractures. Nonfracture interventions included wound suturing, soft tissue reconstruction, and arthroscopic repair of ankle ligamentous structures.

### 2.6 Data collection and outcome measures

Demographic characteristics and clinical data were collected from each participant. Sleep quality was measured via the Pittsburgh Sleep Quality Index (PSQI). The quality of postoperative recovery was assessed via the Quality of Recovery-40 (QoR-40) questionnaire. Anxiety ratings were measured via the Self-rating Anxiety Scale (SAS). The pain catastrophizing scale is used to identify individuals at high risk for pain. After providing informed consent, our research team will provide participants with a standardized postoperative pain documentation form. The participants received specific instructions to precisely record the exact date and time corresponding to the first onset of postoperative pain (VAS score >0) in the surgical area. Patients were followed at 6 h, 12 h, 24 h, 48 h and 72 h after surgery at the bedside. The evaluations and data collection for the aforementioned scales were conducted by dedicated anesthesia nurses trained by our department.

The primary outcome was cumulative sufentanil consumption during the 24 to 48 h postoperatively. The secondary outcomes included the following: (1) Sufentanil consumption at other postoperative time points: 6, 12, 24, 48, and 72 h. (2) duration of analgesia, defined as the interval from nerve block needle withdrawal to the first patient-reported surgical site pain; (3) duration of motor blockade, defined as the interval from needle withdrawal after nerve block until the return of motor function in the patient’s lower leg muscles. (4) Sleep quality assessments on postoperative days 0 (day of surgery), 1, 2, and 3; (5) quality of recovery evaluations conducted on postoperative days 0–3; and (6) incidence of postoperative adverse events.

### 2.7 Sample size calculation

The sample size calculation was performed via PASS 15 software. Given the absence of prior studies investigating liposomal bupivacaine for sciatic and saphenous nerve blocks, we derived parameters from comparable literature on liposomal bupivacaine applications in other peripheral nerve blocks.

The primary outcome measure was total sufentanil consumption 24–48 h after surgery. On the basis of published data (23) demonstrating a mean sufentanil consumption of 37.1 ± 3.3 μg in the liposomal bupivacaine group (Group L) versus 39.1 ± 3.1 μg in the conventional bupivacaine group (Group R), we established the following parameters for calculation: *α* = 0.05, statistical power (1−*β*) = 0.9, and two-sided significance testing for detecting intergroup differences in mean opioid consumption. This initial calculation yielded a minimum requirement of 59 subjects per group. To account for potential attrition (estimated at 20%), the sample size was adjusted upward to 71 participants per group. Consequently, a total cohort of 142 patients was enrolled and randomly allocated to the two treatment arms through computer-generated randomization.

### 2.8 Statistical analysis

All analyses were performed on an intention-to-treat basis. Data normality was measured by the Shapiro–Wilk test. The Levene test was used to verify the homogeneity of variance. Normally distributed continuous variables are presented as the mean ± standard deviation (mean ± SD) and were compared via Student’s *t* test. Nonnormally distributed continuous data are reported as medians (first quartile [Q1]–third quartile [Q3]) and were compared via the Mann–Whitney U test. Categorical data are reported as frequencies (%) and were analyzed via the chi-square test or Fisher’s exact test.

Postoperative repeated measurements of sufentanil dosage were analyzed via generalized estimating equations (GEEs). In this model, the intervention group and time were incorporated as fixed effects, whereas individual patients were treated as random effects to account for intrasubject correlations. The durations of analgesia and motor blockade were analyzed via Kaplan–Meier survival curves, with intergroup differences assessed via the log-rank test. Hazard ratios (HRs) with 95% confidence intervals (CIs) were calculated via Cox regression.

To evaluate postoperative pain patterns in the ropivacaine-treated group during the first 3 days following surgery, we employed the group-based trajectory modeling (GBTM) approach to characterize the pain trajectory profiles. The optimal model configuration was determined through a comprehensive comparison of Bayesian information criterion (BIC) values across multiple candidate models. To systematically identify determinants associated with distinct pain trajectory patterns, we performed univariate logistic regression analysis incorporating variables such as age, sex, type of surgery, and sleep quality. Variables demonstrating statistical associations at *p* < 0.1 in preliminary analyses were subsequently entered into multivariable logistic regression modeling to ascertain independent predictors. The final effect estimates were graphically represented through forest plots, with error bars indicating 95% confidence intervals.

We performed exploratory subgroup analyses to evaluate the consistency of the effect of liposomal bupivacaine across sex and age subgroups. Age was dichotomized at the median of the full cohort. Within each subgroup, outcomes were compared between the two groups using the Mann–Whitney U test or *t*-test, as appropriate. To test for heterogeneity of treatment effects, we constructed regression models that included treatment assignment, the subgroup variable, and their interaction term, and calculated the interaction *p*-value (with *p* < 0.10 considered indicative of potential effect modification).

All the statistical analyses were conducted via R software (version 4.4.2) and SPSS 26.0 software. A *p* value less than 0.05 was considered statistically significant.

## 3 Results

**Figure 1:**
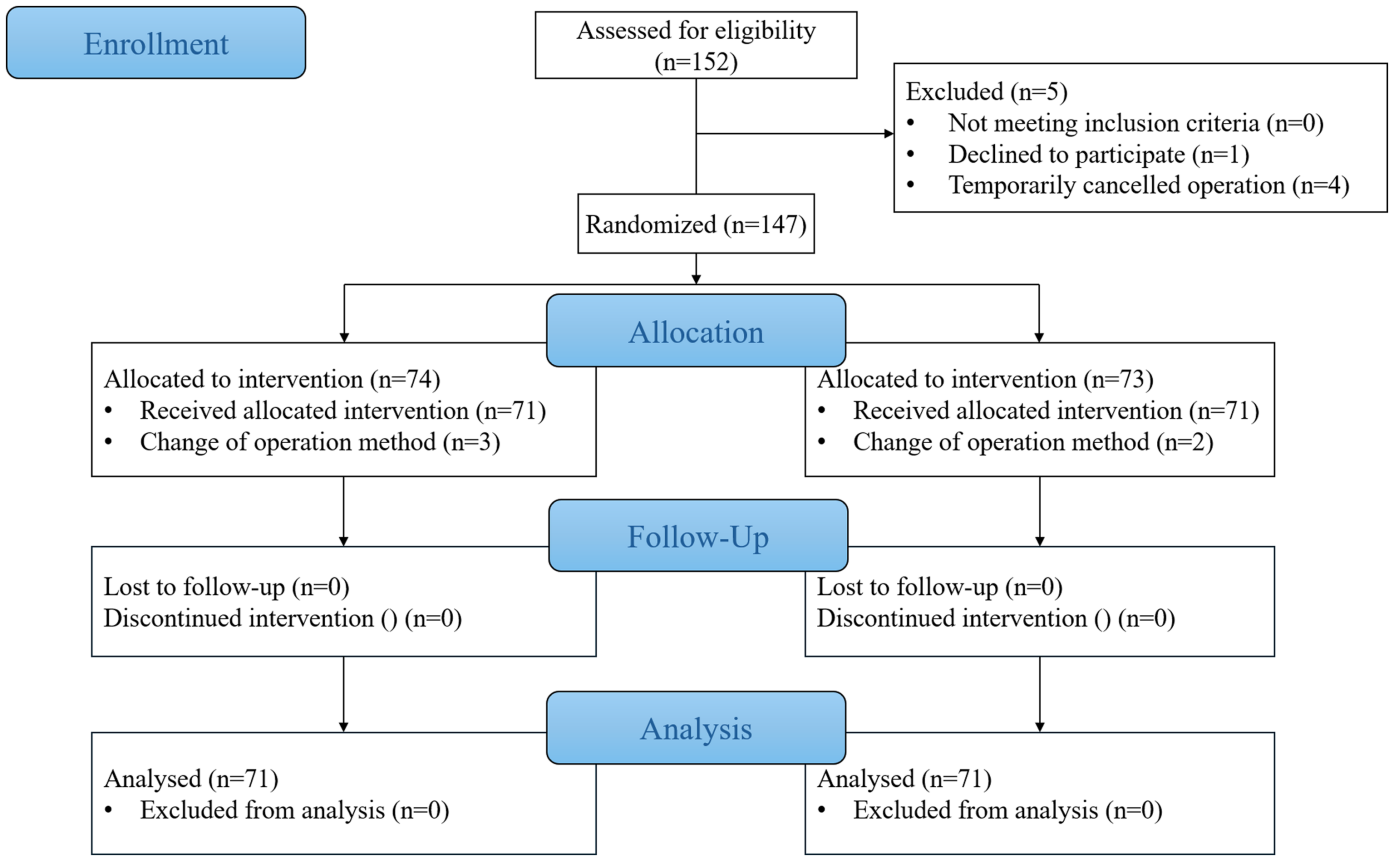
CONSORT flow diagram of the study.

This randomized controlled trial was conducted at Xuzhou Renci Hospital, enrolling 152 patients scheduled for elective foot and ankle surgeries between September 1, 2024 and January 1, 2025. Four participants experienced last-minute surgery cancelations, while one patient refused to sign the informed consent form. Following randomization, protocol deviations occurred in 5 cases: three subjects in Group R and two in Group L did not undergo the intended nerve block procedures. Consequently, 142 patients (71 per group) were ultimately included in the final analysis. Figure 1 presents the complete participant flow diagram.

### 3.1 Baseline demographics and clinical characteristics

No significant differences were observed between the groups regarding age, gender, BMI, ASA classification, operative type, sleep quality, anxiety scale scores, or pain catastrophizing scale scores (Table 1). Furthermore, the groups exhibited comparable intraoperative profiles, including heart rate, mean arterial pressure, operative/anesthesia duration, and vasoactive drug requirements (all *p* > 0.05; Table 1).

**Table 1:** Baseline, surgery and anesthesia characteristics of study patients.

Variables	Group R (*n* = 71)	Group L (*n* = 71)	*P*-value
Age, years, mean (SD)	39.86 (13.39)	37.62 (11.54)	0.288
Gender, *n* (%)			0.737
Female	38 (53.5)	36 (50.7)	
Male	33 (46.5)	35 (49.3)	
BMI, kg/m^2^, mean (SD)	24.74 (2.25)	25.07 (2.72)	0.429
ASA, *n* (%)			0.573
ASAII	65 (91.5)	63 (88.7)	
ASAIII	6 (8.5)	8 (11.3)	
Smoking history, *n* (%)	12 (16.9)	8 (11.3)	0.335
Alcohol history, *n* (%)	11 (15.5)	13 (18.3)	0.654
Operation history, *n* (%)			0.422
Primary Surgery	53 (74.6)	57 (80.3)	
Multiple Surgeries	18 (25.4)	14 (19.7)	
Operative type, *n* (%)			0.473
Fracture	46 (64.8)	50 (70.4)	
Non-fracture injuries	25 (35.2)	21 (29.6)	
Sleep quality, median (Q1–Q3)	3 (1–8)	4 (2–7)	0.548
Anxiety scale, *n* (%)			0.313
Low	11 (15.5)	7 (9.9)	
Median	0	0	
Score of Pain Catastrophizing Scale, median (Q1–Q3)	34 (32–40)	33 (29–38)	0.262
Intraoperative vital signs			
Heart rate, beats·min^−1^, mean (SD)	65.59 (6.44)	67.37 (6.19)	0.096
Mean arterial blood pressure, mmHg, mean (SD)	79.85 (8.79)	82.28 (8.75)	0.100
Vasoactive medications, *n* (%)	12 (16.9)	10 (14.1)	0.643
Ephedrine	5 (7.0)	4 (5.6)	0.731
Phenylephrine	9 (12.7)	7 (9.9)	0.596
Surgery duration, min, median (Q1-Q3)	125 (100–142)	136 (112–147)	0.125
Anesthesia duration, min, median (Q1-Q3)	19 (16–22)	18 (17–22)	0.921

Data represent mean (SD), median (Q1–Q3) or number of subjects (% of column). Sleep quality was measured using Pittsburgh sleep quality index (PSQI); ASA, American society of Anesthesiologists; BMI, body mass index; Anxiety scale was measured using Self-rating anxiety scale (SAS).

### 3.2 Analgesic usage

Statistical analysis using Generalized Estimating Equations (GEEs) revealed significant differences in postoperative sufentanil consumption between Group L and Group R across various time points (*p* < 0.001). The group-time interaction effect did not reach statistical significance. A comparative analysis conducted at specific postoperative intervals revealed that the cumulative consumption of sufentanil in group L was significantly lower than that in group R during the 24–48 h period following surgery (39.27 ± 7.14 μg vs. 34.45 ± 4.72 μg, *p* < 0.001). At the 6 h time point, sufentanil use was comparable between the two groups. However, statistically significant differences between the groups were observed at all subsequent time intervals, including within the first 12 h, 24 h, 48 h, and 72 h postoperatively (all *p* < 0.001) (Table 2).

**Table 2:** Comparison of sufentanil consumption between the two groups.

Time window	Group R (*n* = 71)	Group L (*n* = 71)	Mean Difference (95% CI)	*P*-value
Sufentanil consumption, ug, mean (SD)				
24 h–48 h	39.27 (7.14)	34.45 (4.72)	4.82 (2.81, 6.83)	<0.001
Within 6 h	7.62 (1.30)	7.54 (1.27)	0.09 (−0.34, 0.51)	0.697
Within 12 h	16.80 (2.65)	14.56 (2.24)	2.24 (1.42, 3.06)	<0.001
Within 24 h	50.93 (5.00)	28.96 (3.41)	21.97 (20.55, 23.39)	<0.001
Within 48 h	90.20 (4.70)	63.41 (3.37)	26.79 (25.43, 28.15)	<0.001
Within 72 h	106.83 (4.15)	82.46 (3.45)	24.36 (23.10, 25.63)	<0.001

Data represent mean (SD).

### 3.3 Duration of action of nerve block

**Figure 2:**
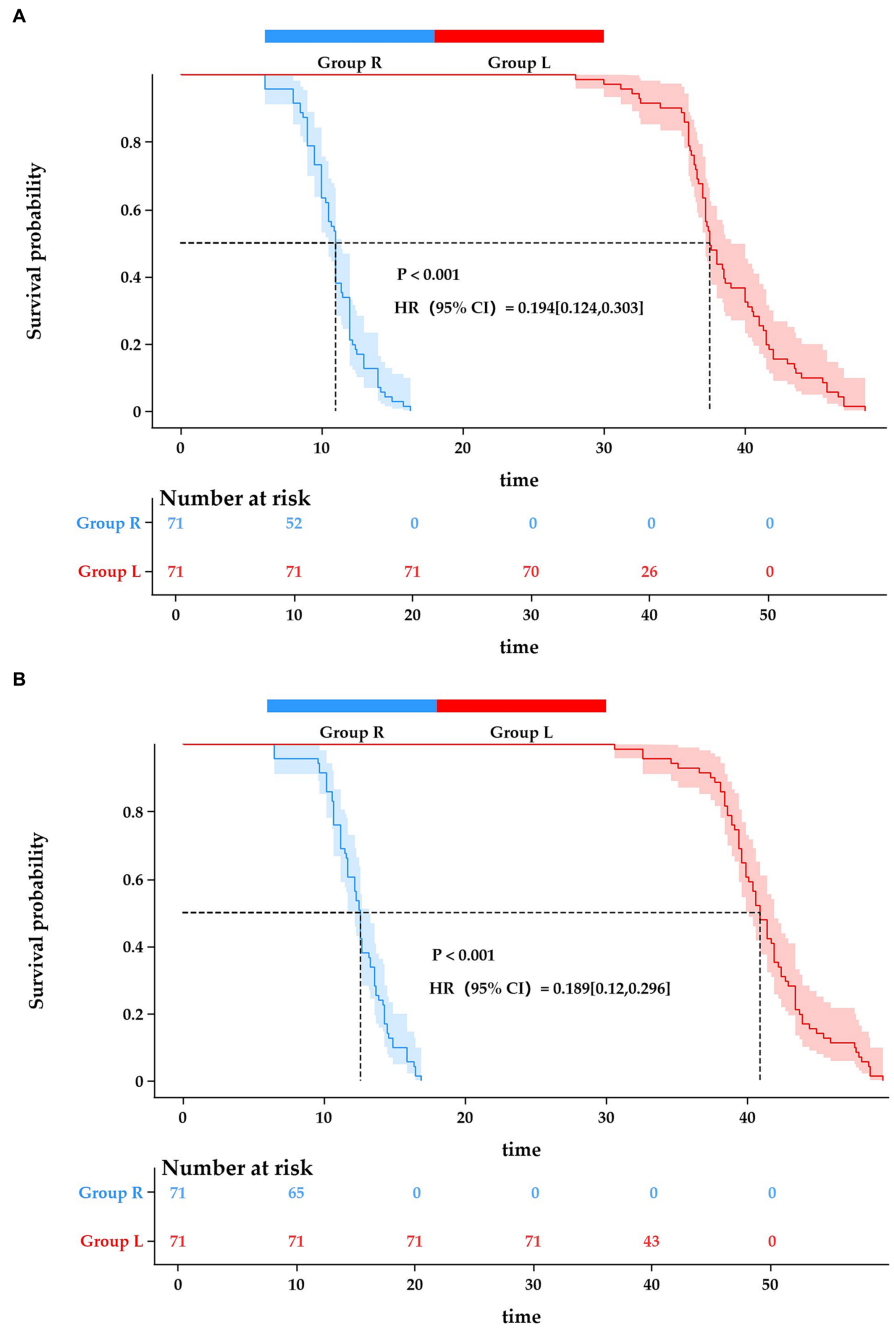
**(A)** Duration of analgesia Kaplan–Meier plot. Kaplan–Meier analysis of analgesia duration curves comparing ropivacaine versus liposomal bupivacaine for postoperative pain management. **(B)** Duration of motor block Kaplan–Meier plot. Kaplan–Meier curves comparing the duration of motor block between Group R and Group L.

The Group L demonstrated significantly prolonged durations of both analgesic block (38.63 ± 4.05 vs. 10.91 ± 2.12 h, *p* < 0.001) and motor block (41.24 ± 3.82 vs. 12.45 ± 2.21 h, *p* < 0.001) compared with the Group R. Kaplan–Meier survival analysis revealed substantial differences in block maintenance between groups for both analgesic (Log-rank test, *p* < 0.001; HR 0.194, 95% CI 0.124–0.303) and motor blockade (Log-rank test, *p* < 0.001; HR 0.189, 95% CI 0.12–0.296) (Figure 2).

### 3.4 Sleep quality and recovery quality

**Figure 3:**
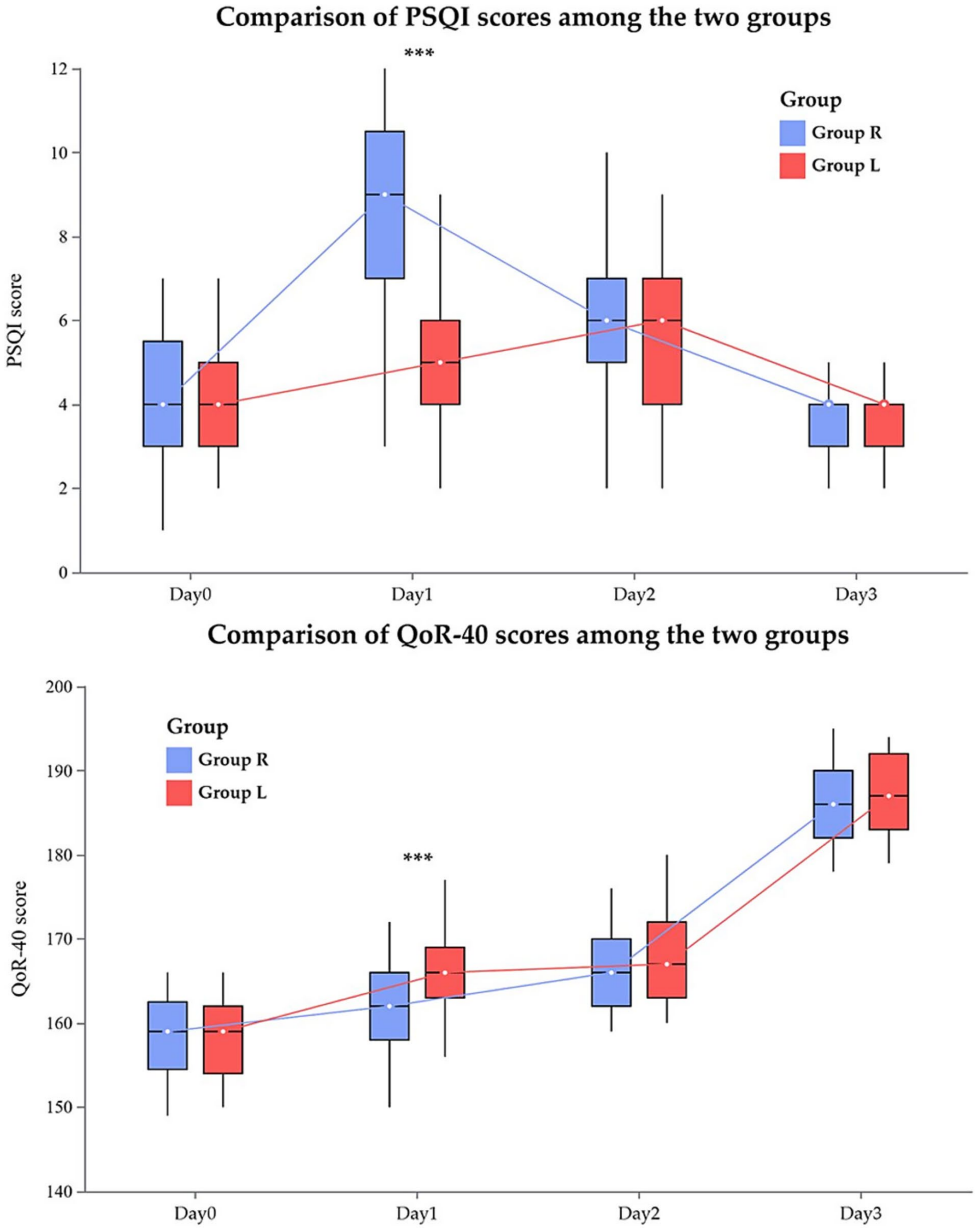
Comparative analysis of sleep quality and recovery quality between two groups at different time points. ***Compared with Group R, *p* < 0.001. Sleep quality was assessed using the Pittsburgh Sleep Quality Index (PSQI), with total scores ranging from 0 to 21, where a score >5 indicates significant sleep disturbance. Postoperative recovery quality was evaluated using the 40-item Quality of Recovery (QoR-40) questionnaire, which yields a total score ranging from 40 to 200. A total score <160 was considered indicative of poor postoperative recovery, with higher scores reflecting better recovery quality.

Figure 3 illustrates the differences in postoperative sleep quality, recovery quality, and their trends between the two groups: Compared to Group R, Group L exhibited a significantly lower Pittsburgh Sleep Quality Index (PSQI) score on postoperative day 1 (5.00 [4.00, 6.00] vs. 9.00 [7.00, 11.00]; *p* < 0.001). On postoperative day 1, the QoR-40 score was significantly lower in Group L compared to Group R (166.00 [163.00, 169.00] vs. 162.00 [158.00, 166.00]; *p* < 0.001). Except at postoperative day 1, the two groups showed no significant differences in recovery quality or sleep quality at any other time points.

### 3.5 Adverse events

The incidence of postoperative nausea and vomiting (PONV) was significantly lower in Group L than in Group R (4.2% vs. 14.1%; *p* = 0.042), corresponding to an absolute risk reduction of 9.9% (95% confidence interval [CI]: 0.5 to 19.2%) and a number needed to treat (NNT) of 10. Similarly, the incidence of dizziness was significantly reduced in Group L compared with Group R (5.6% vs. 16.9%; *p* = 0.034), with an absolute risk difference of 11.3% (95% CI: 1.0 to 21.5%) and an NNT of 9. Postoperative complications—including infections, pyrexia, wound exudation, abdominal distension, somnolence, pruritus, respiratory depression, and urinary retention—occurred at low rates (all < 5%), with no significant differences observed between the groups (*p* > 0.05 for all comparisons) (Table 3).

**Table 3:** Incidence of adverse reactions.

Adverse event	Group R (*n* = 71)	Group L (*n* = 71)	*P*-value	Absolute risk difference (95% CI)
PONV, *n* (%)	10 (14.1)	3 (4.2)	0.042	9.9% (0.5 to 19.2%)
Dizziness, *n* (%)	12 (16.9)	4 (5.6)	0.034	11.3% (1.0 to 21.5%)
Fever, *n* (%)	2 (2.8)	1 (1.4)	1.000	
Abdominal distension, *n* (%)	3 (4.2)	2 (2.8)	1.000	
Reflux, *n* (%)	2 (2.8)	0 (0)	0.496	
Urinary retention, *n* (%)	3 (4.2)	1 (1.4)	0.620	
Constipation, *n* (%)	2 (2.8)	3 (4.2)	1.000	
diarrhea, *n* (%)	3 (4.2)	2 (2.8)	1.000	
Infection, *n* (%)	0 (0)	2 (2.8)	0.496	
Bandage seep through, *n* (%)	2 (2.8)	1 (1.4)	1.000	
Sedation, *n* (%)	1 (1.4)	0 (0)	1.000	
Pruritus, *n* (%)	4 (5.6)	3 (4.2)	1.000	
Respiratory depression, *n* (%)	0 (0)	0 (0)	1.000	

Data represent number of subjects (% of column).

### 3.6 Establishing a latent trajectory model and conducting multivariable logistic regression analysis of pain trajectories

**Figure 4:**
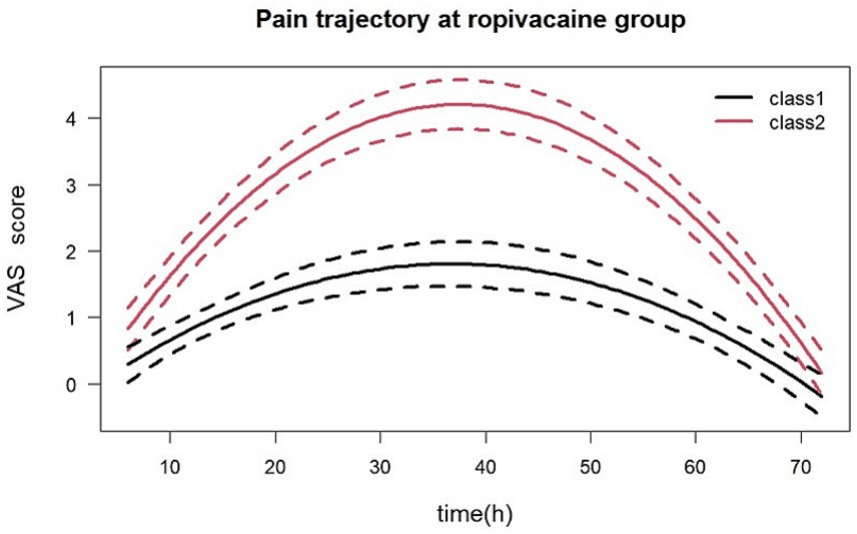
Pain trajectory in the ropivacaine group identified by latent class trajectory modeling. Patients were classified into two distinct trajectory patterns based on postoperative pain scores over time. Class 1 (black trajectory) represents a “remaining-low” class, characterized by consistently low pain levels throughout the observation period. Class 2 (red trajectory) represents a “rapidly-increasing” class, characterized by initially low pain scores followed by a steep increase over time.

Latent class trajectory modeling identified two distinct postoperative pain trajectories in the ropivacaine group: a rapidly-increasing class (red trajectory) and a remaining-low class (black trajectory) (Figure 4). Using the identified trajectory classes as the dependent variable, univariate logistic regression analyses were performed to assess associations between trajectory membership and demographic/clinical variables including age, gender, ASA physical status classification, surgical type, and other relevant parameters.

**Figure 5:**
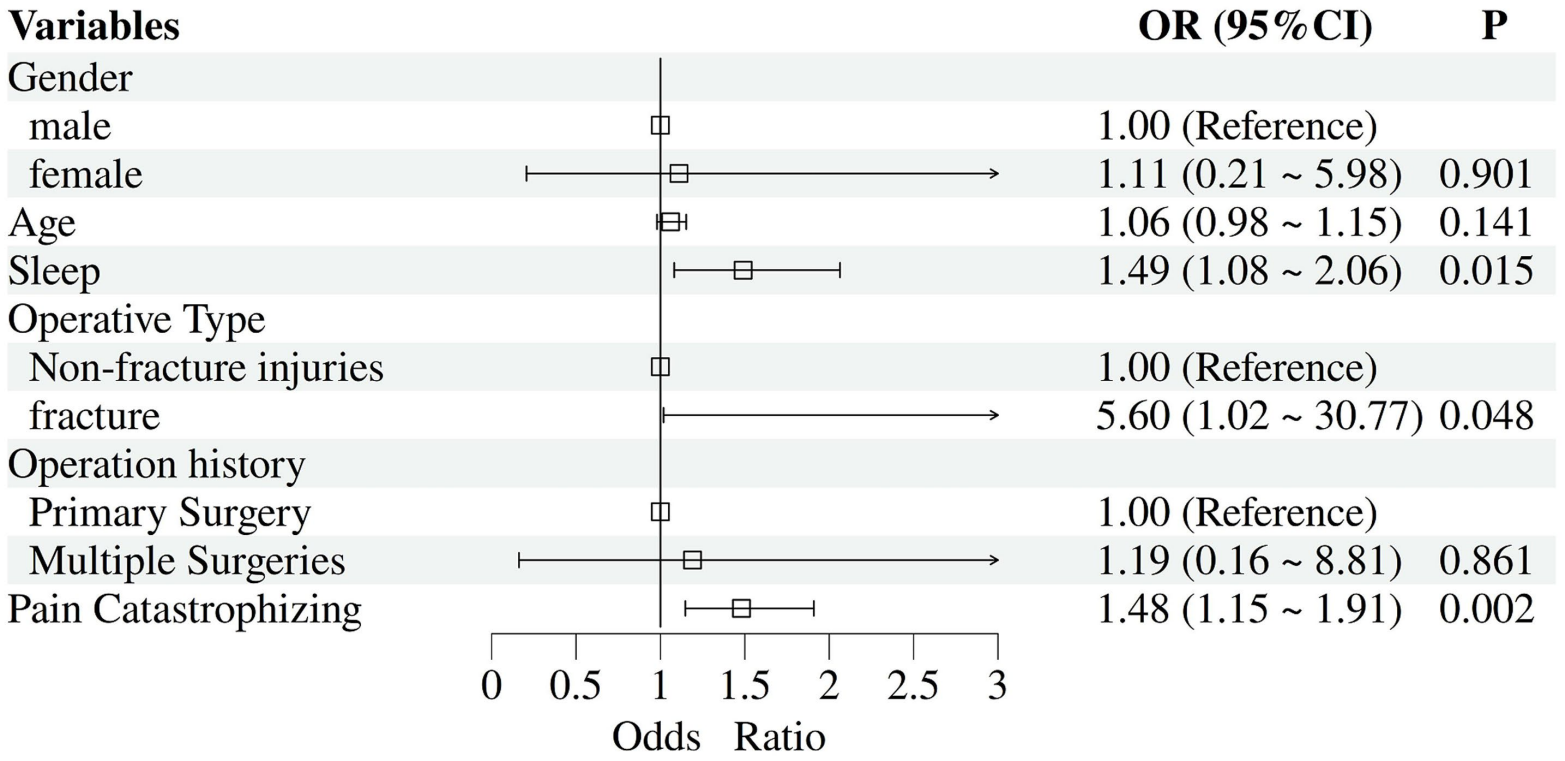
Multivariable logistic regression analysis of pain trajectory development.

The univariate analysis revealed significant associations (*p* < 0.1) with surgical type, history of multiple surgeries, age, gender, sleep quality, and Pain Catastrophizing Scale scores. Multivariable logistic regression analysis demonstrated that surgical type (adjusted odds ratio OR = 5.60; 95% CI: 1.02–30.77), sleep quality (OR = 1.49; 95% CI: 1.08–2.06), and Pain Catastrophizing Scale scores (OR = 1.48; 95% CI: 1.15–1.91) emerged as independent predictors of pain trajectory classification (Figure 5).

### 3.7 Exploratory subgroup analysis

To investigate the potential heterogeneity in the treatment effect of liposomal bupivacaine, we performed exploratory subgroup analyses stratified by sex and age. Across all predefined subgroups (male, female, age ≤ 35 years, age > 35 years), the liposomal bupivacaine group consistently demonstrated superior outcomes compared to the control group in terms of reduced sufentanil consumption, prolonged duration of sensory block, and improved sleep quality and postoperative recovery quality (all within-subgroup *p* < 0.05). However, interaction analyses revealed no statistically significant differences in the treatment effect (liposomal bupivacaine vs. control) between sex-based (all *P* for interaction >0.10) or age-based (all *P* for interaction >0.10) subgroups (Supplementary Tables 1, 2).

## 4 Discussion

In this single-center, double-blind, randomized controlled trial, we demonstrated that, compared with ropivacaine, liposomal bupivacaine significantly reduced postoperative opioid consumption, prolonged the duration of nerve blockade, and improved both postoperative sleep quality and recovery quality. Furthermore, preoperative sleep quality, type of surgery, and pain catastrophizing scale scores were predictive of the development of a rapidly increasing pain trajectory.

Foot and ankle surgery frequently results in significant postoperative pain (24). As a major postoperative complication, optimizing postoperative pain management is paramount for facilitating patient recovery and mitigating the risk of pain-related adverse outcomes (25). Over 80% of patients experience postoperative pain of varying intensity, with peak pain severity typically persisting for 24–72 h (26). Owing to their inherent pharmacological profiles, conventionally used local anesthetics often fail to provide adequate coverage throughout this critical period of maximal postoperative pain (13). Extending the duration of peripheral nerve blocks is pivotal for achieving superior postoperative analgesia, which in turn enhances the quality of postoperative recovery—a cornerstone of Enhanced Recovery After Surgery (ERAS) protocols. Dexamethasone as an adjuvant can prolong the analgesic duration; however, its use in patients with hyperglycemia remains controversial (27, 28). Continuous nerve blocks provide analgesia comparable to that of liposomal bupivacaine but carry risks of infection and catheter dislodgement, along with higher costs (29). Liposomal bupivacaine has emerged as an active area of research in recent years and, owing to its reported prolonged duration of analgesia, represents a novel option for perioperative pain management. In this study, the administration of liposomal bupivacaine via popliteal sciatic and saphenous nerve blocks significantly reduced sufentanil consumption during the 24 to 48 h postoperative period. Furthermore, our results revealed that sufentanil requirements within the first 6 h after surgery were comparable to those observed with ropivacaine. However, from 6 h onward and continuing until postoperative day 3, the group receiving liposomal bupivacaine demonstrated a significantly decreased need for sufentanil. As previously reported (30), liposomal bupivacaine transversus abdominis plane (TAP) block significantly reduces total opioid consumption within the first 72 h following cesarean delivery. Similarly, our study demonstrated that it effectively reduces opioid consumption in patients undergoing foot and ankle surgery. Furthermore, in our study, the application of liposomal bupivacaine significantly prolonged the duration of analgesia (38.63 ± 4.05 h). This result confirms the efficacy of liposomal bupivacaine in extending analgesic duration among patients undergoing foot and ankle surgery. Although the observed duration did not reach the marketed claim of 72 h (31), it nevertheless resulted in a statistically significant prolongation of the analgesic effect compared with the control group receiving ropivacaine (10.91 ± 2.12 h). Liposomal bupivacaine employs multivesicular liposome technology to encapsulate bupivacaine within lipid vesicles. Upon administration, liposomes provide sustained drug release through sequential disruption of their vesicular layers (32), which accounts for their prolonged analgesic efficacy in foot and ankle surgeries.

The sciatic nerve bifurcates superior to the popliteal fossa into the tibial nerve and the common peroneal nerve, which predominantly innervate the posterior leg and plantar foot and the anterolateral leg and dorsum of the foot, respectively. Sciatic nerve blockade in the popliteal fossa is widely employed for patients undergoing lower limb and ankle procedures (33). However, a single sciatic nerve block does not provide analgesic coverage for the medial ankle or lower leg. Combining this technique with a saphenous nerve block effectively addresses this gap. The saphenous nerve, a pure sensory cutaneous branch of the femoral nerve, primarily innervates the skin of the medial aspect of the lower leg and ankle. A study by Maagaard et al. (34) demonstrated that a popliteal sciatic nerve block combined with a saphenous nerve block provides optimal analgesia for patients undergoing foot and ankle surgery. In our cohort, the findings support the efficacy of this combined regional anesthetic technique. It appears to be a safe and effective perioperative analgesic strategy for this specific patient population.

Liu et al. (18) reported that transversus abdominis plane (TAP) block with liposomal bupivacaine resulted in greater recovery at 24 h postcesarean delivery than bupivacaine alone. Consistent with these findings, our study demonstrated that the liposomal bupivacaine group achieved significantly higher QoR-40 scores on postoperative day 1 than did the ropivacaine group. Furthermore, our findings indicate that liposomal bupivacaine, relative to ropivacaine, was associated with superior sleep quality on the first postoperative day. In terms of adverse events, the incidence of observed adverse reactions such as infection, fever, and diarrhea during the hospitalization period following treatment was low in both groups, with no unexpected adverse events reported. These findings suggest that the safety profiles of liposomal bupivacaine and ropivacaine are comparable. Notably, patients in the L group exhibited an approximate 9.9% absolute risk reduction in postoperative nausea and vomiting (PONV) and an 11.3% reduction in dizziness compared to those in the R group, a reduction that can be attributed to the decreased consumption of sufentanil and its associated opioid-related adverse events. The presentation of these absolute risk differences further substantiates the clinical value of liposomal bupivacaine in mitigating adverse postoperative outcomes.

Findings from our exploratory subgroup analyses indicated that the favorable effects of liposomal bupivacaine were consistent across subgroups stratified by sex and age. Despite previous reports suggesting that age and sex can impact drug metabolism (35–37), our interaction analysis revealed that these demographic factors did not significantly modify the treatment effect. Given the exploratory nature of these analyses, however, this observation requires confirmation in adequately powered, prospective studies.

The study by Shankarappa et al. demonstrated (38) that the combination of site 1 sodium channel blockers with quaternary lidocaine derivatives (QLDs) could extend sciatic nerve blockade by 8- to 10-fold. However, QLDs alone induced significant myotoxicity, and even with combination therapy, residual muscle toxicity remained a concern. Their findings highlighted the need for a cautious risk–benefit assessment of such prolonged blocks, questioning whether the duration of blockade justifies the potential cellular-level risks. In contrast, the motor blockade achieved with liposomal bupivacaine in the present study, which lasted approximately 41.24 ± 3.82 h, was not associated with the severe local tissue toxicity concerns reported with QLDs. Current clinical evidence supports the favorable safety profile of liposomal bupivacaine in peripheral nerve blocks (15, 16). The extended duration observed with liposomal bupivacaine is attributed to its multivesicular liposome technology (18, 19), representing a clinically viable approach to achieving long-acting blockade that may circumvent the myotoxicity concerns associated with chemical modifications such as QLDs, as discussed by Shankarappa et al. Furthermore, future investigations should focus on the influence of genetic factors on block duration to facilitate the development of individualized analgesic strategies.

While liposomal bupivacaine significantly prolongs postoperative analgesia, it is important to acknowledge that sciatic nerve blockade, particularly with this formulation (motor block duration: 41.24 ± 3.82 h), can lead to pronounced motor weakness—a factor that warrants careful consideration in clinical practice (39). Early postoperative ambulation is a cornerstone of Enhanced Recovery After Surgery (ERAS) protocols, and evidence suggests (40) that mobilization on postoperative day one or even the day of surgery can significantly shorten hospital stays and accelerate functional recovery. Prolonged motor blockade may therefore conflict with ERAS principles, potentially increasing the risk of patient falls and placing an additional burden on nursing staff. Consequently, we advocate for an individualized approach to the application of this technique. For patient populations requiring strict postoperative immobilization—such as those undergoing complex joint reconstruction or requiring postoperative casting—this prolonged blockade can provide superior analgesia while avoiding pain-related limitations to immobilization. In these patients, with the assistance of family members or ward nurses, ankle pump exercises and gentle joint flexion and extension can be performed. These activities may help reduce swelling in the operated joint, prevent ankle stiffness and adhesions, decrease the incidence of deep vein thrombosis (DVT) in the lower extremities (41), and ultimately enhance functional recovery, recovery quality, and patient satisfaction. Conversely, for patients on a clear ERAS pathway anticipated to ambulate within 6 to 12 h post-surgery, this approach should be used with caution. In such cases, adjustments to local anesthetic concentration may be considered to strike a balance between effective analgesia and the preservation of motor function. Future research should focus on optimizing drug dosages or exploring combination therapies that can shorten the duration of motor blockade while preserving the analgesic benefits. In our study, latent trajectory modeling revealed two distinct pain trajectories within the ropivacaine group. These findings demonstrate heterogeneity in postoperative pain progression among patients receiving ropivacaine nerve block. Further multivariate logistic regression analysis revealed that surgical type, sleep quality, and pain catastrophizing scale scores were independently associated with distinct pain trajectory patterns. Specifically, patients who underwent fracture-related surgery, those reporting poorer sleep quality, and those with higher pain catastrophizing scale scores were more likely to develop a rapidly increasing pain trajectory. Preoperative sleep disorders can induce patient anxiety, which increases sensitivity to pain and subsequently exacerbates postoperative pain intensity (42). Pain catastrophizing refers to exaggerated and negative mental rumination concerning actual or anticipated pain experiences. It represents a maladaptive psychological response closely linked to pain perception (43). Moreover, persistent pain catastrophizing has been shown to exacerbate patients’ pain experience and diminish analgesic efficacy (44), which aligns with the findings of our study. Identifying high-risk factors for rapidly increasing postoperative pain trajectories and implementing personalized interventions such as liposomal bupivacaine nerve blocks holds greater clinical significance for improving postoperative pain management outcomes.

This study has several limitations. First, at the time of study design, our objective was to compare two clinically relevant local anesthetic strategies widely used in practice: liposomal bupivacaine (133 mg) versus standard ropivacaine (50 mg). It is important to emphasize that these doses were selected based on routine clinical dosages recommended by their respective prescribing information and clinical guidelines. Consequently, this study represents a comparison of two clinical strategies rather than a pharmacological equipotent comparison of the formulations themselves. We acknowledge that the 133 mg dose of liposomal bupivacaine and the 50 mg dose of ropivacaine are not matched for potency or pharmacokinetics, complicating the attribution of the observed analgesic benefits specifically to the liposomal formulation rather than the higher equipotent dose. Future studies should consider employing pharmacologically equivalent doses to more accurately isolate the clinical benefits attributable to the formulation itself. Furthermore, while the control group in the present study received 50 mg of ropivacaine, clinicians in real-world settings may adjust the dose or employ adjuncts based on individual patient needs. Therefore, future research should investigate whether our findings remain applicable under conditions involving adjunctive agents or higher ropivacaine doses. Second, the duration of analgesia and motor blockade was recorded based on patient self-assessment, particularly during nighttime hours, which may have introduced a degree of measurement bias and potential overestimation. Although more frequent clinical assessments or portable objective monitoring tools could theoretically provide more precise data, their implementation in routine clinical practice poses significant challenges, including disruption of patient sleep, limitations in personnel resources, and a lack of portable devices suitable for continuous monitoring. Therefore, future research should focus on developing patient-friendly continuous monitoring technologies. In the context of the present study, this measurement bias may have led to a systematic overestimation of block durations. Nevertheless, because the same assessment method was applied across all groups, any bias is likely to be non-differential, and thus the relative differences between groups remain informative. Third, although Xuzhou Renci Hospital is a Grade A tertiary orthopedic hospital and the anesthesiologists possess specialized expertise in peripheral nerve blocks for foot and ankle surgery, we cannot guarantee that every block was performed with perfect technical consistency throughout the study period. This variability in technical execution may have influenced the consistency and efficacy of the nerve blocks. Fourth, it should be acknowledged that latent class trajectory modeling was applied only to the control group, which limits our ability to directly compare pain trajectories between the two groups. The trajectory analysis in this study should therefore be considered exploratory and hypothesis-generating, aimed at uncovering underlying patterns of pain heterogeneity under conventional analgesia, rather than a formal evaluation of the intervention effect. Future studies should consider applying trajectory modeling to larger interventional cohorts to further elucidate how liposomal bupivacaine may differentially modulate analgesic outcomes across distinct pain trajectory subtypes.

## 5 Conclusion

Liposomal bupivacaine for popliteal sciatic and saphenous nerve blocks significantly reduced postoperative opioid consumption, prolonged block duration, and improved postoperative sleep quality and functional recovery. As a novel technique for providing postoperative analgesia in patients undergoing foot and ankle surgery, it demonstrated acceptable safety and efficacy under the conditions of this study. Preoperative sleep quality, type of foot and ankle surgery, and Pain Catastrophizing Scale scores serve as independent predictors of pain trajectories, which could help identify patients more likely to benefit from this specific technique.

## Data Availability

The raw data supporting the conclusions of this article will be made available by the authors, without undue reservation.

## References

[1] Welck MJ Hayes T Pastides P Khan W Rudge B. Stress fractures of the foot and ankle. Injury. (2017) 48:1722–6. doi: 10.1016/j.injury.2015.06.015, 26412591

[2] Hembree WC Tarka MC Pasternack JB Mathew SE Guyton GP. What's new in foot and ankle surgery. J Bone Joint Surg Am. (2023) 105:737–43. doi: 10.2106/jbjs.22.01382, 36888693

[3] Meara JG Leather AJ Hagander L Alkire BC Alonso N Ameh EA . Global surgery 2030: evidence and solutions for achieving health, welfare, and economic development. Lancet. (2015) 386:569–624. doi: 10.1016/s0140-6736(15)60160-x, 25924834

[4] Palmen LN Belt M van Hooff ML Witteveen AGH. Outcome measures after foot and ankle surgery: a systematic review. Foot Ankle Surg. (2025) 31:654–71. doi: 10.1016/j.fas.2025.02.005, 40021414

[5] Hannig KE Hauritz RW Bjørn S Jensen HI Henriksen CW Jessen C . Pain relief after major ankle and hindfoot surgery with repetitive peripheral nerve blocks: a feasibility study. Acta Anaesthesiol Scand. (2023) 67:1266–72. doi: 10.1111/aas.14289, 37280182

[6] Dillane D Ramadi A Nathanail S Dick BD Bostick G Chan K . Elective surgery in ankle and foot disorders-best practices for management of pain: a guideline for clinicians. Can J Anaesth. (2022) 69:1053–67. doi: 10.1007/s12630-022-02267-4, 35581524

[7] El-Boghdadly K Levy NA Fawcett WJ Knaggs RD Laycock H Baird E . Peri-operative pain management in adults: a multidisciplinary consensus statement from the Association of Anaesthetists and the British pain society. Anaesthesia. (2024) 79:1220–36. doi: 10.1111/anae.16391, 39319373

[8] Ivascu R Torsin LI Hostiuc L Nitipir C Corneci D Dutu M. The surgical stress response and anesthesia: a narrative review. J Clin Med. (2024) 13:3017. doi: 10.3390/jcm13103017, 38792558 PMC11121777

[9] Russell G Lightman S. The human stress response. Nat Rev Endocrinol. (2019) 15:525–34. doi: 10.1038/s41574-019-0228-0, 31249398

[10] Liu Y Xiao S Yang H Lv X Hou A Ma Y . Postoperative pain-related outcomes and perioperative pain management in China: a population-based study. Lancet Reg Health West Pac. (2023) 39:100822. doi: 10.1016/j.lanwpc.2023.100822, 37927993 PMC10625022

[11] Aparisi Gómez MP Aparisi F Guglielmi G Bazzocchi A. Particularities on anatomy and normal postsurgical appearances of the ankle and foot. Radiol Clin North Am. (2023) 61:281–305. doi: 10.1016/j.rcl.2022.10.013, 36739146

[12] Clark IC Allman RD Rogers AL Goda TS Smith K Chanas T . Multimodal pain management protocol to decrease opioid use and to improve pain control after thoracic surgery. Ann Thorac Surg. (2022) 114:2008–14. doi: 10.1016/j.athoracsur.2022.03.059, 35430217

[13] Schubert AK Wiesmann T Dinges HC. Measures to prolong duration of sensory block after regional anaesthesia. Curr Opin Anaesthesiol. (2023) 36:103–8. doi: 10.1097/aco.0000000000001204, 36326074

[14] Ilfeld BM Eisenach JC Gabriel RA. Clinical effectiveness of liposomal bupivacaine administered by infiltration or peripheral nerve block to treat postoperative pain. Anesthesiology. (2021) 134:283–344. doi: 10.1097/aln.0000000000003630, 33372949

[15] Nedeljkovic SS Kett A Vallejo MC Horn J-L Carvalho B Bao X . Transversus abdominis plane block with liposomal bupivacaine for pain after cesarean delivery in a multicenter, randomized, double-blind, controlled trial. Anesth Analg. (2020) 131:1830–9. doi: 10.1213/ane.0000000000005075, 32739962 PMC7643795

[16] Patel MA Gadsden JC Nedeljkovic SS Bao X Zeballos JL Yu V . Brachial plexus block with liposomal bupivacaine for shoulder surgery improves analgesia and reduces opioid consumption: results from a multicenter, randomized, double-blind, controlled trial. Pain Med. (2020) 21:387–400. doi: 10.1093/pm/pnz103, 31150095 PMC12481196

[17] Ganguly K Van Helmond N Friedman A Ahmad R Bowen F III Shersher DD . Liposomal bupivacaine versus bupivacaine and dexamethasone intercostal nerve blocks for robotic thoracic surgery: a randomized clinical trial. Cureus. (2024) 16:e62085. doi: 10.7759/cureus.62085, 38989396 PMC11236214

[18] Liu HH Qiu D Xu DR Yang JJ Teng PL. Recovery quality of transversus abdominis plane block with liposomal bupivacaine after cesarean delivery: a randomized trial. J Clin Anesth. (2024) 99:111608. doi: 10.1016/j.jclinane.2024.111608, 39265467

[19] Nguyen A Grape S Gobbetti M Albrecht E. The postoperative analgesic efficacy of liposomal bupivacaine versus long-acting local anaesthetics for peripheral nerve and field blocks: a systematic review and meta-analysis, with trial sequential analysis. Eur J Anaesthesiol. (2023) 40:624–35. doi: 10.1097/eja.0000000000001833, 37038770 PMC10860892

[20] Wu L Xi C Lei G Li H Yin Y Wan M . Noninferiority of 0.25% versus 0.375% ropivacaine in popliteal sciatic and saphenous nerve blocks for analgesia after foot and ankle surgery: a randomized self-paired noninferiority trial. Drug Des Devel Ther. (2025) 19:4093–104. doi: 10.2147/dddt.S508528, 40416797 PMC12101467

[21] Xia Z Liu Y Lu Z Gan J Yu M Olsen K . The impact of product quality attributes on in vivo performance of bupivacaine multivesicular liposomes. Drug Deliv Transl Res. (2025) 15:3268–80. doi: 10.1007/s13346-025-01806-y, 40035967

[22] Hadzic A Abikhaled JA Harmon WJ. Impact of volume expansion on the efficacy and pharmacokinetics of liposome bupivacaine. Local Reg Anesth. (2015) 8:105–11. doi: 10.2147/lra.S88685, 26673040 PMC4676620

[23] Chevrollier GS Klinger AL Green HJ Gastanaduy MM Johnston WF Vargas HD . Liposomal bupivacaine Transversus abdominis plane blocks in laparoscopic colorectal resections: a single-institution randomized controlled trial. Dis Colon Rectum. (2023) 66:322–30. doi: 10.1097/dcr.0000000000002346, 35849756

[24] Short AJ Ghosh M Jin R Chan VWS Chin KJ. Intermittent bolus versus continuous infusion popliteal sciatic nerve block following major foot and ankle surgery: a prospective randomized comparison. Reg Anesth Pain Med. (2019):rapm-2018-100301. doi: 10.1136/rapm-2018-100301, 31570495

[25] Schou NK Svensson LGT Cleemann R Andersen JH Mathiesen O Maagaard M. The efficacy and safety of ankle blocks for foot and ankle surgery: a systematic review with meta-analysis and trial sequential analysis. Foot Ankle Surg. (2024) 30:355–65. doi: 10.1016/j.fas.2024.02.015, 38492998

[26] Koschmieder KC Funcke S Shadloo M Pinnschmidt HO Greiwe G Fischer M . Validation of three nociception indices to predict immediate postoperative pain before emergence from general anaesthesia: a prospective double-blind, observational study. Br J Anaesth. (2023) 130:477–84. doi: 10.1016/j.bja.2022.11.024, 36609057

[27] Maagaard M Albrecht E Mathiesen O. Prolonging peripheral nerve block duration: current techniques and future perspectives. Acta Anaesthesiol Scand. (2025) 69:e70010. doi: 10.1111/aas.70010, 40000382 PMC11860723

[28] Katerenchuk V Ribeiro EM Batista AC. Impact of intraoperative dexamethasone on perioperative blood glucose levels: systematic review and meta-analysis of randomized trials. Anesth Analg. (2024) 139:490–508. doi: 10.1213/ane.0000000000006933, 39151135

[29] Panchamia JK Smith HM Sanchez-Sotelo J Vinsard PA Duncan CM Njathi-Ori CW . Randomized clinical trial comparing mixed liposomal bupivacaine vs. continuous catheter for interscalene block during shoulder arthroplasty: a comparison of analgesia, patient experience, and cost. J Shoulder Elb Surg. (2025) 34:2178–89. doi: 10.1016/j.jse.2024.09.051, 39622357

[30] Habib AS Nedeljkovic SS Horn JL Smiley RM Kett AG Vallejo MC . Randomized trial of transversus abdominis plane block with liposomal bupivacaine after cesarean delivery with or without intrathecal morphine. J Clin Anesth. (2021) 75:110527. doi: 10.1016/j.jclinane.2021.110527, 34626927

[31] Chahar P Cummings KC III. Liposomal bupivacaine: a review of a new bupivacaine formulation. J Pain Res. (2012) 5:257–64. doi: 10.2147/jpr.S27894 23049275 PMC3442744

[32] Chan TCW Wong JSH Wang F Fang CX Yung CS Chan MTH . Addition of liposomal bupivacaine to standard bupivacaine versus standard bupivacaine alone in the supraclavicular brachial plexus block: a randomized controlled trial. Anesthesiology. (2024) 141:732–44. doi: 10.1097/aln.0000000000005035, 38696340 PMC11389883

[33] Karmakar MK Reina MA Sivakumar RK Areeruk P Pakpirom J Sala-Blanch X. Ultrasound-guided subparaneural popliteal sciatic nerve block: there is more to it than meets the eyes. Reg Anesth Pain Med. (2021) 46:268–75. doi: 10.1136/rapm-2020-101709, 33077429

[34] Maagaard M Funder KS Schou NK Penny JØ Toquer P Laigaard J . Combined dexamethasone and dexmedetomidine as adjuncts to popliteal and saphenous nerve blocks in patients undergoing surgery of the foot or ankle: a randomized, blinded, placebo-controlled clinical trial. Anesthesiology. (2024) 140:1165–75. doi: 10.1097/aln.0000000000004977, 38489226

[35] Wasilczuk AZ Rinehart C Aggarwal A Stone ME Mashour GA Avidan MS . Hormonal basis of sex differences in anesthetic sensitivity. Proc Natl Acad Sci USA. (2024) 121:e2312913120. doi: 10.1073/pnas.2312913120, 38190526 PMC10801881

[36] Valodara AM Sr KJ. Sexual dimorphism in drug metabolism and pharmacokinetics. Curr Drug Metab. (2019) 20:1154–66. doi: 10.2174/1389200220666191021094906, 31631817

[37] Al Harbi MK Alshaghroud SM Aljahdali MM Ghorab FA Baba F Al Dosary R . Regional anesthesia for geriatric population. Saudi J Anaesth. (2023) 17:523–32. doi: 10.4103/sja.sja_424_23, 37779559 PMC10540989

[38] Shankarappa SA Sagie I Tsui JH Chiang HH Stefanescu C Zurakowski D . Duration and local toxicity of sciatic nerve blockade with coinjected site 1 sodium-channel blockers and quaternary lidocaine derivatives. Reg Anesth Pain Med. (2012) 37:483–9. doi: 10.1097/AAP.0b013e31826125b3, 22914659 PMC3428729

[39] Moosa F Allan A Bedforth N. Regional anaesthesia for foot and ankle surgery. BJA Educ. (2022) 22:424–31. doi: 10.1016/j.bjae.2022.07.005, 36304911 PMC9596281

[40] Frassanito L Vergari A Nestorini R Cerulli G Placella G Pace V . Enhanced recovery after surgery (ERAS) in hip and knee replacement surgery: description of a multidisciplinary program to improve management of the patients undergoing major orthopedic surgery. Musculoskelet Surg. (2020) 104:87–92. doi: 10.1007/s12306-019-00603-4, 31054080

[41] Wainwright TW Gill M McDonald DA Middleton RG Reed M Sahota O . Consensus statement for perioperative care in total hip replacement and total knee replacement surgery: enhanced recovery after surgery (ERAS(®)) society recommendations. Acta Orthop. (2020) 91:3–19. doi: 10.1080/17453674.2019.1683790, 31663402 PMC7006728

[42] Nowakowski S Levy-Meeks ME Dawson DB Meers JM Stout-Aguilar JS Kilic GS . Association of preoperative sleep pattern with posthysterectomy pain: a pilot study. J Clin Sleep Med. (2020) 16:1901–8. doi: 10.5664/jcsm.8730, 32776870 PMC8034220

[43] Petrini L Arendt-Nielsen L. Understanding pain catastrophizing: putting pieces together. Front Psychol. (2020) 11:603420. doi: 10.3389/fpsyg.2020.603420, 33391121 PMC7772183

[44] Bierke S Petersen W. Influence of anxiety and pain catastrophizing on the course of pain within the first year after uncomplicated total knee replacement: a prospective study. Arch Orthop Trauma Surg. (2017) 137:1735–42. doi: 10.1007/s00402-017-2797-5, 28965133

