# Supplementary material for: Liposomal bupivacaine for popliteal sciatic and saphenous nerve blocks in patients undergoing foot and ankle surgery: a single-center, double-blind, randomized controlled trial
Source: Front Med (Lausanne). 2026 Mar 3;13:1753354. doi: 10.3389/fmed.2026.1753354 (PMC13040354; doi:10.3389/fmed.2026.1753354)
Supplement: Supplementary file 1 [file Table_1.docx]

**Supplemental Table 1**. Subgroup Analysis of Primary Outcomes

| Outcome | Subgroup | Group R (n=71) |  | Group L  (n=71) | Between-Group *P*-value¹ | *P*-value for Interaction² |
| --- | --- | --- | --- | --- | --- | --- |
| Opioids consumption, ug, mean (SD) |  |  |  |  |  |  |
| Within 6 h | Sex |  |  |  |  | 0.229 |
|  | Male (n=68) | 7.73 (1.33) |  | 7.37 (1.29) | 0.266 |  |
|  | Female (n=74) | 7.53 (1.29) |  | 7.69(1.26) | 0.573 |  |
|  | Age³ |  |  |  |  | 0.711 |
|  | ≤35 years (n=74) | 7.69 (1.26) |  | 7.67 (1.33) | 0.950 |  |
|  | >35 years (n=68) | 7.56 (1.36) |  | 7.38(1.21) | 0.567 |  |
| Within 12 h | Sex |  |  |  |  | 0.577 |
|  | Male | 16.88 (2.60) |  | 14.40 (2.33) | ＜0.001 |  |
|  | Female | 16.74 (2.74) |  | 14.72(2.17) | ＜0.001 |  |
|  | Age |  |  |  |  | 0.302 |
|  | ≤35 years | 16.69 (2.64) |  | 14.85 (2.41) | 0.003 |  |
|  | >35 years | 16.92 (2.70) |  | 14.22(2.00) | ＜0.001 |  |
| Within 24 h | Sex |  |  |  |  | 0.976 |
|  | Male | 50.36 (5.30) |  | 28.40 (3.06) | ＜0.001 |  |
|  | Female | 51.42 (4.74) |  | 29.50(3.68) | ＜0.001 |  |
|  | Age |  |  |  |  | 0.261 |
|  | ≤35 years | 51.63 (3.58) |  | 28.85 (2.98) | ＜0.001 |  |
|  | >35 years | 50.25 (6.04) |  | 29.09(3.91) | ＜0.001 |  |
| Within 48 h | Sex |  |  |  |  | 0.897 |
|  | Male | 90.45 (4.68) |  | 63.74 (3.50) | ＜0.001 |  |
|  | Female | 89.97 (4.77) |  | 63.08(3.26) | ＜0.001 |  |
|  | Age |  |  |  |  | 0.541 |
|  | ≤35 years | 90.54 (4.93) |  | 63.33 (3.24) | ＜0.001 |  |
|  | >35 years | 89.86 (4.52) |  | 63.50(3.57) | ＜0.001 |  |
| Within 72 h | Sex |  |  |  |  | 0.935 |
|  | Male | 107.00 (4.42) |  | 82.57 (3.58) | ＜0.001 |  |
|  | Female | 106.68 (3.95) |  | 82.36(3.37) | ＜0.001 |  |
|  | Age |  |  |  |  | 0.133 |
|  | ≤35 years | 107.06 (4.10) |  | 81.79 (3.42) | ＜0.001 |  |
|  | >35 years | 106.61 (4.24) |  | 83.28(3.36) | ＜0.001 |  |

**Supplemental Table 2****.** Subgroup Analysis of Other Outcome Measures

| Outcome | Subgroup | Group R  (n=71) |  | Group L  (n=71) | Between-Group *P*-value¹ | *P*-value for Interaction² |
| --- | --- | --- | --- | --- | --- | --- |
| QoR-40 score |  |  |  |  |  |  |
| Day1 | Sex |  |  |  |  | 0.382 |
|  | Male (n=68) | 162 (160.5-166) |  | 168 (163-172) | ＜0.001 |  |
|  | Female (n=74) | 160 (154-165) |  | 166 (163-168) | ＜0.001 |  |
|  | Age³ |  |  |  |  | 0.971 |
|  | ≤35 years (n=74) | 161 (157-166) |  | 166 (163-169) | ＜0.001 |  |
|  | >35 years (n=68) | 162 (159-165) |  | 166 (165-169) | ＜0.001 |  |
| Day2 |  |  |  |  |  |  |
|  | Sex |  |  |  |  | 0.990 |
|  | Male | 166 (162.5-171) |  | 166 (163-172) | 0.231 |  |
|  | Female | 166 (162-170) |  | 167 (163-172) | 0.143 |  |
|  | Age |  |  |  |  | 0.704 |
|  | ≤35 years | 166 (162-170) |  | 166 (163-172) | 0.130 |  |
|  | >35 years | 166 (162-171) |  | 167 (163-172) | 0.235 |  |
| PSQI score |  |  |  |  |  |  |
| Day1 | Sex |  |  |  |  | 0.786 |
|  | Male | 9 (7-10) |  | 4 (4-5) | ＜0.001 |  |
|  | Female | 9 (7-11) |  | 5 (4-6) | ＜0.001 |  |
|  | Age |  |  |  |  | 0.274 |
|  | ≤35 years | 9 (7-11) |  | 4 (3-5) | ＜0.001 |  |
|  | >35 years | 8 (7-10) |  | 5 (4-6) | ＜0.001 |  |
| Day2 | Sex |  |  |  |  | 0.904 |
|  | Male | 6 (4-7) |  | 5 (4-7) | 0.666 |  |
|  | Female | 6 (5-7) |  | 6 (4-7) | 0.899 |  |
|  | Age |  |  |  |  | 0.262 |
|  | ≤35 years | 6 (4-6) |  | 6 (5-7) | 0.239 |  |
|  | >35 years | 6 (5-7) |  | 5.5 (4-6.75) | 0.078 |  |
| Durations of motor block (h) | Sex |  |  |  |  | 0.880 |
|  | Male | 12.91 (1.74) |  | 41.75 (4.17) | ＜0.001 |  |
|  | Female | 12.06 (2.51) |  | 40.74 (3.44) | ＜0.001 |  |
|  | Age |  |  |  |  | 0.248 |
|  | ≤35 years | 12.18 (2.00) |  | 41.55 (3.88) | ＜0.001 |  |
|  | >35 years | 12.71 (2.40) |  | 40.86 (3.78) | ＜0.001 |  |
| Durations of analgesic block (h) | Sex |  |  |  |  | 0.960 |
|  | Male | 11.33 (1.89) |  | 39.05 (4.50) | ＜0.001 |  |
|  | Female | 10.55 (2.26) |  | 38.21 (3.58) | ＜0.001 |  |
|  | Age |  |  |  |  | 0.142 |
|  | ≤35 years | 10.54 (1.84) |  | 39.02 (4.02) | ＜0.001 |  |
|  | >35 years | 11.27 (2.32) |  | 38.15 (4.10) | ＜0.001 |  |

¹*P* values for between-group comparisons indicate differences in outcomes between the liposomal bupivacaine group and the control group within each specified subgroup.

²*P* values for interaction were used to assess whether the treatment effects (i.e., between-group differences) varied significantly across levels of subgroup variables (sex or age group).

Age³ subgroups were defined by the median age of the entire cohort (35 years).

Data are presented as mean (SD) or median (Q1–Q3).
